# The Evolution of Gallic Acid in Aged White Tea and Its Potential Anti‐Aging Mechanisms: An Integrated Study Combining Network Pharmacology and Computer Simulation

**DOI:** 10.1002/fsn3.71246

**Published:** 2025-11-29

**Authors:** Yanming Tuo, Xiaofeng Lu, Qingying Song, Ruiqi Huang, Jiapeng Huang, Huan Sun, Ningkai Liao, Yutao Shi, Liangyu Wu, Jinke Lin, Yunfei Hu

**Affiliations:** ^1^ College of Horticulture Fujian Agriculture and Forestry University Fuzhou China; ^2^ College of tea and Food Sciences Wuyi University Wuyishan China; ^3^ Anxi College of Tea Science Fujian Agriculture and Forestry University Fuzhou China

**Keywords:** aging, gallic acid, molecular docking, molecular dynamics simulation, network pharmacology, tea

## Abstract

Aging, characterized by a gradual decline in physiological function, is a major risk factor for chronic diseases. White tea, one of China's traditional tea types, exhibits various health benefits due to its unique chemical composition, with its anti‐aging potential drawing increasing attention. Gallic acid (GA), one of the important bioactive components in white tea, possesses antioxidant and anti‐inflammatory properties, but its anti‐aging mechanisms remain unclear. In this study, high‐performance liquid chromatography was used to analyze the evolution of GA content and to determine the antioxidant capacity of aged white tea. Results revealed that the GA content increased with storage time, accompanied by a corresponding enhancement in the antioxidant potency composite index (APC). Network pharmacology predicted 40 potential anti‐aging targets of GA, and protein–protein interaction network analysis identified six key targets (MAOA, PTGS2, BCL2, APP, IGF1R, SERPINE1). Functional enrichment analysis indicated that the anti‐aging effects of GA are mediated through multiple pathways, particularly those related to oxidative stress. Molecular docking results demonstrated that GA could bind effectively to the six key targets via hydrogen bonding and hydrophobic interactions. Furthermore, molecular dynamics simulations confirmed the binding stability of GA with MAOA, PTGS2, and BCL2. This study systematically elucidates the evolution of GA in aged white tea and its potential anti‐aging mechanisms, providing a theoretical basis for the development of GA and aged white tea as functional anti‐aging additives.

## Graphical abstract

In this study, HPLC was used to analyze the evolution of GA content and to determine the antioxidant capacity of aged white tea. Results revealed that the GA content increased with storage time, accompanied by a corresponding enhancement in the antioxidant potency composite index. Network pharmacology and protein–protein interaction network analysis identified six key targets (MAOA, PTGS2, BCL2, APP, IGF1R, SERPINE1). Furthermore, molecular docking and molecular dynamics simulations confirmed the binding stability of GA with MAOA, PTGS2, and BCL2.

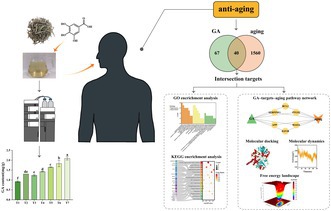

## 1 Introduction

With improvements in quality of life and advancements in healthcare, life expectancy has been steadily increasing worldwide. It is estimated that by 2050, the proportion of the global population aged over 60 will nearly double (Cho and Stout‐Delgado 2020). Aging, a progressive decline in physiological function with advancing age, is a major risk factor for numerous chronic diseases, including cardiovascular disorders, cancer, and osteoporosis (Montégut et al. 2024; Zhou et al. 2022). The incidence of these age‐related diseases is expected to rise significantly in the coming decades (de Magalhães et al. 2011). Therefore, delaying the aging process and preventing the onset of aging‐associated diseases have become pressing public health goals.

However, aging is a complex biological process involving chronic disorders from the molecular to the systemic level (Du et al. 2024). Studies have shown that DNA damage caused by various stressors, such as oxidative stress, ultraviolet radiation, and chemotherapeutic drugs, is a major cause of cellular aging (Ou and Schumacher 2018). Accordingly, multiple anti‐aging strategies have been developed, among which dietary restriction, appropriate exercise, and gene therapy are considered important approaches (Hood et al. 2018; Soultoukis and Partridge 2016; Tian et al. 2019). However, dietary and exercise interventions are often difficult to sustain in the general population, and gene therapies remain unapproved and are still under investigation. This has led to increased interest in small‐molecule drugs as feasible anti‐aging candidates (Partridge et al. 2020). Nevertheless, although some drugs have shown certain effectiveness in anti‐aging, issues such as side effects and individual variability in efficacy remain prominent (Dolan et al. 2024). In recent years, natural products have drawn considerable attention due to their high safety profiles and minimal side effects, prompting widespread investigation into their anti‐aging potential.

White tea, one of the six traditional tea categories in China, has attracted global interest owing to its unique processing methods and broad spectrum of health benefits. Increasing evidence has demonstrated that white tea possesses antioxidant, anti‐inflammatory, and anti‐aging properties (Somavanshi et al. 2022). Although it undergoes minimal processing compared to other teas, white tea contains relatively high levels of polyphenolic compounds (Zhang et al. 2022). Among these, gallic acid (GA), a phenolic acid and a widely distributed secondary metabolite in plants, stands out as a key polyphenol in tea with potent antioxidant and anti‐inflammatory activities (Yan et al. 2018). Previous research has reported that GA content increases with storage time in black tea (Wu et al. 2023). Additionally, White Tip Silver Needle white tea has been shown to mitigate aging symptoms in mice induced by D‐galactose/lipopolysaccharide (Li et al. 2021). Mice treated with aqueous extracts of white tea exhibited reduced epidermal thickness and increased levels of collagen and elastic fibers (Lee et al. 2014).

Moreover, GA has been found to decrease inflammatory cytokines and oxidative stress markers in rat embryonic fibroblasts (REF), reduce the proportion of senescent REF cells in the G_0_/G_1_ phase, and promote progression into the G_2_/M phase. GA also inhibits apoptosis by suppressing caspase‐9 activity (Rahimifard et al. 2020). In mice, GA significantly improved TAC‐induced cardiac dysfunction and alleviated pathological alterations, including myocardial hypertrophy, inflammation, and oxidative stress (Yan et al. 2018). A study by Eunson Hwang and colleagues demonstrated that GA exerted photoprotective effects by inhibiting ROS and MMP‐1 activity, thereby reducing UVB‐induced skin dryness, thickening, and wrinkle formation, while promoting the expression of type I procollagen and elastin (Hwang et al. 2014).

Although both white tea and GA have demonstrated anti‐aging effects, the underlying molecular mechanisms remain unclear. Network pharmacology, an emerging discipline that integrates systems biology and computational biology, offers a systematic approach to identifying the potential targets of GA and understanding its role within biological networks (Noor et al. 2023). Molecular docking techniques can predict the binding patterns and affinities between drug molecules and target proteins (Tao et al. 2019). While molecular dynamics simulations further validate docking results and provide insights into the dynamic interactions at the molecular level (Wu et al. 2022). These computational tools represent powerful strategies for elucidating the anti‐aging mechanisms of GA.

In this study, the content of GA in white tea samples from 2017 to 2023 was quantified to investigate its evolution during aging. Network pharmacology, molecular docking, and molecular dynamics simulations were employed to systematically explore the potential anti‐aging mechanisms of GA. The findings of this study provide a theoretical foundation for the development of anti‐aging white tea products and the biomedical applications of GA derived from aged white tea.

## 2 Materials and Methods

### 2.1 Tea Samples and Chemicals

**FIGURE 1:**
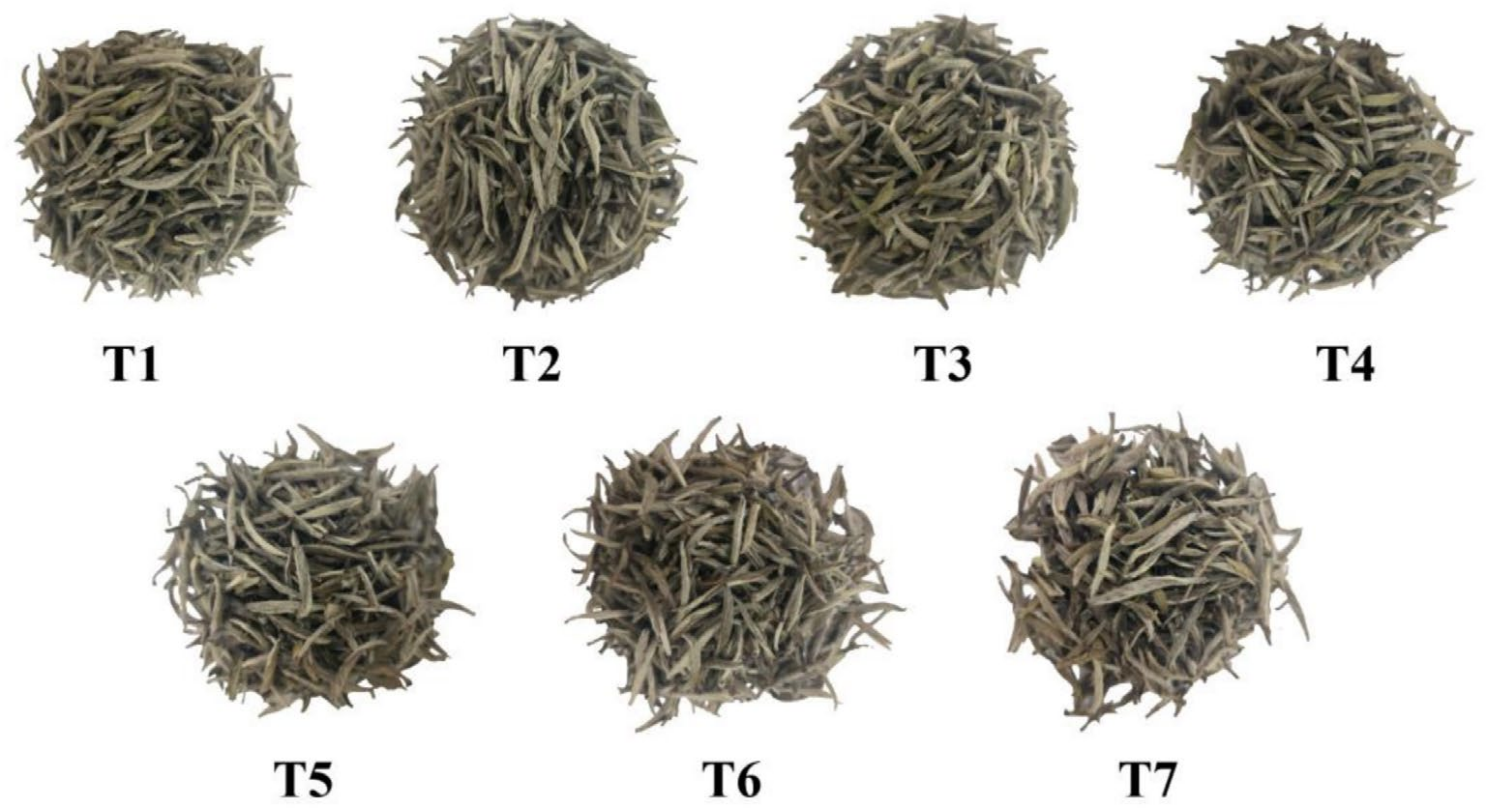
Appearance of white tea samples with seven different storage years.

All white tea samples were obtained from Xiyang Tea Co. Ltd. (Fuding, Fujian, China). As shown in Figure 1, seven white tea samples harvested in different years—2023, 2022, 2021, 2020, 2019, 2018, and 2017—were labeled as T1 through T7, respectively. All fresh leaves were sourced from the same tea plantation, derived from *Camellia sinensis* (L.) O. Kuntze cv. Fuding Dahao, and classified as single‐bud grade.

The processing procedure was as follows: fresh single‐bud leaves were immediately transported to the withering room, evenly spread on bamboo trays (2–3 cm thick), and subjected to 48 h of natural indoor withering under controlled conditions (20°C–25°C, 60%–70% relative humidity). The withered leaves were then dried in a dryer at 60°C for 3 h to yield the final white tea samples. For aging, all samples were produced using the same protocol and stored in sealed aluminum foil bags under uniform conditions (dry, ventilated warehouse; relative humidity ≤ 50%; ambient temperature ≤ 35°C). Archived samples were collected in June 2023 from the company's inventory and stored at −20°C until analysis.

Standard GA (> 99%), HPLC‐grade methanol, and glacial acetic acid were purchased from Sigma‐Aldrich (Shanghai, China), and HPLC‐grade acetonitrile was obtained from Merck (Darmstadt, Germany).

### 2.2 Quantification of GA

Sample preparation was performed according to the Chinese National Standard (GB/T 8313–2018). The GA content was quantified using a Shimadzu LC‐20A high‐performance liquid chromatography (HPLC) system (Shimadzu, Tokyo, Japan) equipped with a Phenomenex C18 column (5 μm, 4.6 mm × 250 mm). The mobile phases were as follows: phase A—9.0% acetonitrile and 2% glacial acetic acid; phase B—80% acetonitrile. The flow rate was 1.0 mL/min, column temperature was maintained at 35°C, detection wavelength was 278 nm, and the injection volume was 10 μL.

**FIGURE 2:**
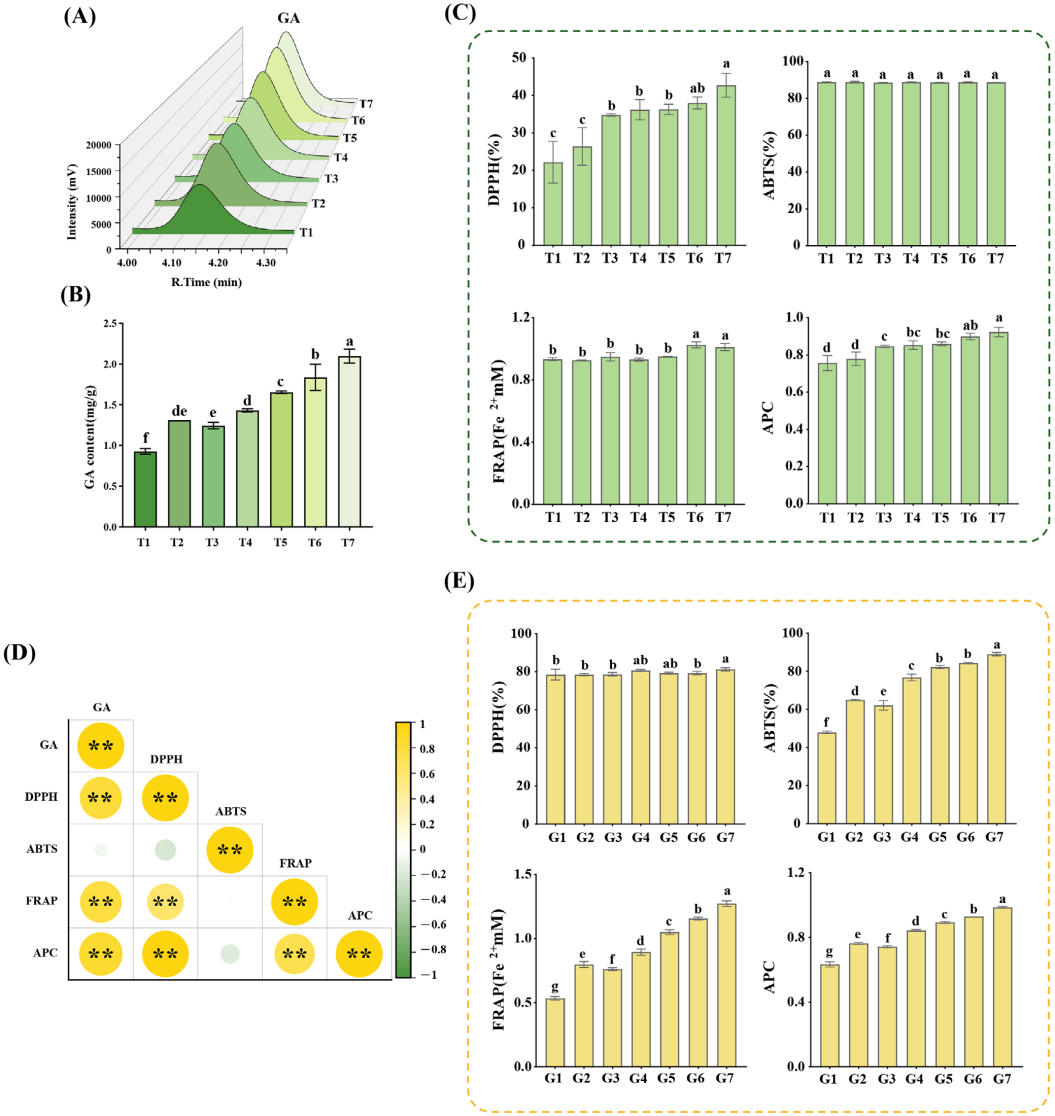
(A) HPLC chromatograms of GA in white tea samples with seven different storage years. (B) Variation in GA content among the seven storage years. (C) Changes in antioxidant capacity of white tea samples with different storage years (*n* = 3; different letters indicate significant differences at *p* < 0.05). (D) Correlation between antioxidant capacity and GA content in white tea samples (*n* = 3; pearson correlation; ** indicate significant differences at *p* < 0.01). (E) Changes in antioxidant capacity of GA standard solutions at different concentrations (*n* = 3; different letters indicate significant differences at *p* < 0.05).

The gradient elution program was as follows: 0–10 min, 0% B; 10–23 min, linear increase to 30% B; 23–27 min, held at 30% B; 27–30 min, return to initial conditions. A blank solvent sample was injected after every 10 samples to monitor system stability. Chromatograms were generated using Origin 2022 (Figure 2A).

### 2.3 Antioxidant Activity Assay

Tea extracts were prepared as described by (Wang, Liang, et al. 2023). Briefly, 1.0 g of ground tea sample was mixed with 80 mL of boiling water in a conical flask and extracted in a 100°C water bath for 45 min with vigorous shaking every 10 min. The extract was immediately filtered while hot and then cooled to room temperature before being diluted to a final volume of 100 mL. Based on the measured GA content in T1–T7, GA standard solutions were prepared at concentrations of 18.53, 26.20, 24.80, 28.54, 33.00, 36.80, and 42.00 μg/mL, respectively, and labeled as G1–G7 in increasing order. Both tea infusions and GA solutions were subjected to antioxidant assays, including the antiradical activity against 2,2‐diphenyl‐1‐picrylhydrazyl (DPPH), the scavenging activity of 2,2′‐azino‐bis (3‐ethylbenzothiazoline‐6‐sulfonic acid) (ABTS), and ferric reducing antioxidant power (FRAP). These assays were conducted using commercial kits (Shanghai Yuanye Bio‐Technology Co. Ltd., Shanghai, China) in accordance with the manufacturer's instructions. All antioxidant measurements were performed in biological triplicates.

The antioxidant potency composite index (APC) was calculated based on the method of Wang, Wang, et al. (2023) using the following formula:

APC=DDmax+AAmax+FFmax3

where *D*, *A*, and *F* represent the DPPH, ABTS, and FRAP values of each sample, and *D*_max_, *A*_max_, and *F*_max_ are the respective maximum values across all samples.

### 2.4 Prediction and Screening of GA Anti‐Aging Targets

The Canonical SMILES of GA was retrieved from PubChem (https://pubchem.ncbi.nlm.nih.gov/). Target prediction was performed using four databases: SwissTargetPrediction, SEA Search Server, Super‐PRED, and CODD‐Pred. The predicted targets were merged, and duplicates were removed. The GA–target network was constructed using Cytoscape 3.9.1.

Aging‐related targets were identified using the keyword “aging” in GeneCards (https://www.genecards.org/), OMIM (https://omim.org/), and MalaCards (https://www.malacards.org/). For GeneCards, targets with a relevance score ≥ 5.5 were selected. All aging‐related targets were combined and deduplicated.

Gene names were standardized using bioDBnet (https://biodbnet‐abcc.ncifcrf.gov/db/db2db.php). Overlapping targets between GA and aging datasets were identified by intersection analysis and visualized with a Venn diagram using the Metware Cloud platform (https://cloud.metware.cn/).

### 2.5 Construction and Analysis of Key Target–Protein–Protein Interaction (PPI) Network

The overlapping targets were imported into the STRING database (http://string‐db.org/) to construct a PPI network using “ *Homo sapiens* ” as the species and a minimum interaction score of 0.4. Other parameters were set to default (Szklarczyk et al. 2023). The resulting network was visualized in Cytoscape 3.9.1. The CytoNCA plugin was used to calculate degree centrality (DC), betweenness centrality (BC), and closeness centrality (CC). The top 10 targets from each metric were compared, and overlapping targets were identified as key anti‐aging targets of GA (Tang et al. 2014).

### 2.6 GO and KEGG Pathway Enrichment Analysis

Gene Ontology (GO) and Kyoto Encyclopedia of Genes and Genomes (KEGG) pathway enrichment analyses were conducted using the “clusterProfiler” package with a significance threshold of *p* < 0.05. GO functional analysis predicted gene functions across three categories: biological process (BP), cellular component (CC), and molecular function (MF). While KEGG pathway enrichment analysis identified key pathways associated with the anti‐ aging targets of GA. Visualization of the enrichment results was carried out via Metware Cloud (https://cloud.metware.cn/) and Gene Denovo (https://www.genedenovo.com/).

### 2.7 Molecular Docking

The SDF file of GA was downloaded from PubChem. Three‐dimensional structures of MAOA (PDB ID: 2Z5Y), PTGS2 (AlphaFold ID: AF‐P35354‐F1), BCL2 (PDB ID: 6GL8), APP (AlphaFold ID: AF‐P05067‐F1), IGF1R (PDB ID: 5FXS), and SERPINE1 (PDB ID: 1LJ5) were obtained from the UniProt database (Ko et al. 2022). Molecular docking was performed using AutoDock Vina 1.2.5 (Eberhardt et al. 2021), and results were analyzed and visualized using Discovery Studio 2019.

### 2.8 Molecular Dynamics (MD) Simulation

MD simulations of MAOA–GA, PTGS2–GA, and BCL2–GA complexes were carried out using GROMACS 2020.6. The AMBER99SB force field and SPC water model were applied. The system was simulated at 300 K for 50 ns. Energy minimization was performed using the steepest descent method, followed by equilibration. Binding free energy was calculated using both MM/GBSA and MM/PBSA methods (Lagarias et al. 2020), and results were visualized using Xmgrace 5.1.25 and Origin 2024. Free energy landscapes were constructed and analyzed based on root mean square deviation (RMSD), radius of gyration (Rg), and Gibbs free energy.

### 2.9 Data Analysis

One‐way analysis of variance (ANOVA) was performed using IBM SPSS 25.0 (IBM SPSS, Armonk, NY, USA). Bar charts were generated using GraphPad Prism 9.

## 3 Results

### 3.1 Variation in GA Content and Antioxidant Activity in White Tea Samples of Different Storage Years

In this study, the GA content in seven white tea samples (T1–T7) from different production years was systematically quantified. As shown in Figure 2B, GA content exhibited a significant upward trend with increasing storage time. Specifically, the lowest GA content was found in T1 (0.93 mg/g) and the highest in T7 (2.10 mg/g), with values for T2‐T6 being 1.31, 1.24, 1.43, 1.65, and 1.84 mg/g, respectively.

Antioxidant activities of the tea samples are illustrated in Figure 2C. DPPH values increased significantly with aging time, while FRAP showed a significant increase only in T6 and T7. No significant variation was observed in ABTS across samples. However, the APC showed a positive trend with increasing storage years. Correlation analysis between GA content and antioxidant indices showed strong positive correlations between GA levels and DPPH, FRAP, and APC (Figure 2D).

As shown in Figure 2E, antioxidant activities of GA standard solutions (G1–G7), prepared to match the GA concentrations found in T1–T7, demonstrated significant differences, especially at the G7 concentration. Both ABTS and FRAP values increased with rising GA concentration, mirroring the trend observed in tea samples. Similarly, APC increased significantly with higher GA concentrations.

Collectively, these results indicate that GA content in white tea increases significantly with storage time and that antioxidant capacity improves accordingly. The findings from both tea infusions and corresponding GA solutions provide supportive evidence for the potential anti‐aging effects of GA.

### 3.2 Screening of Potential Anti‐Aging Targets of GA

**FIGURE 3:**
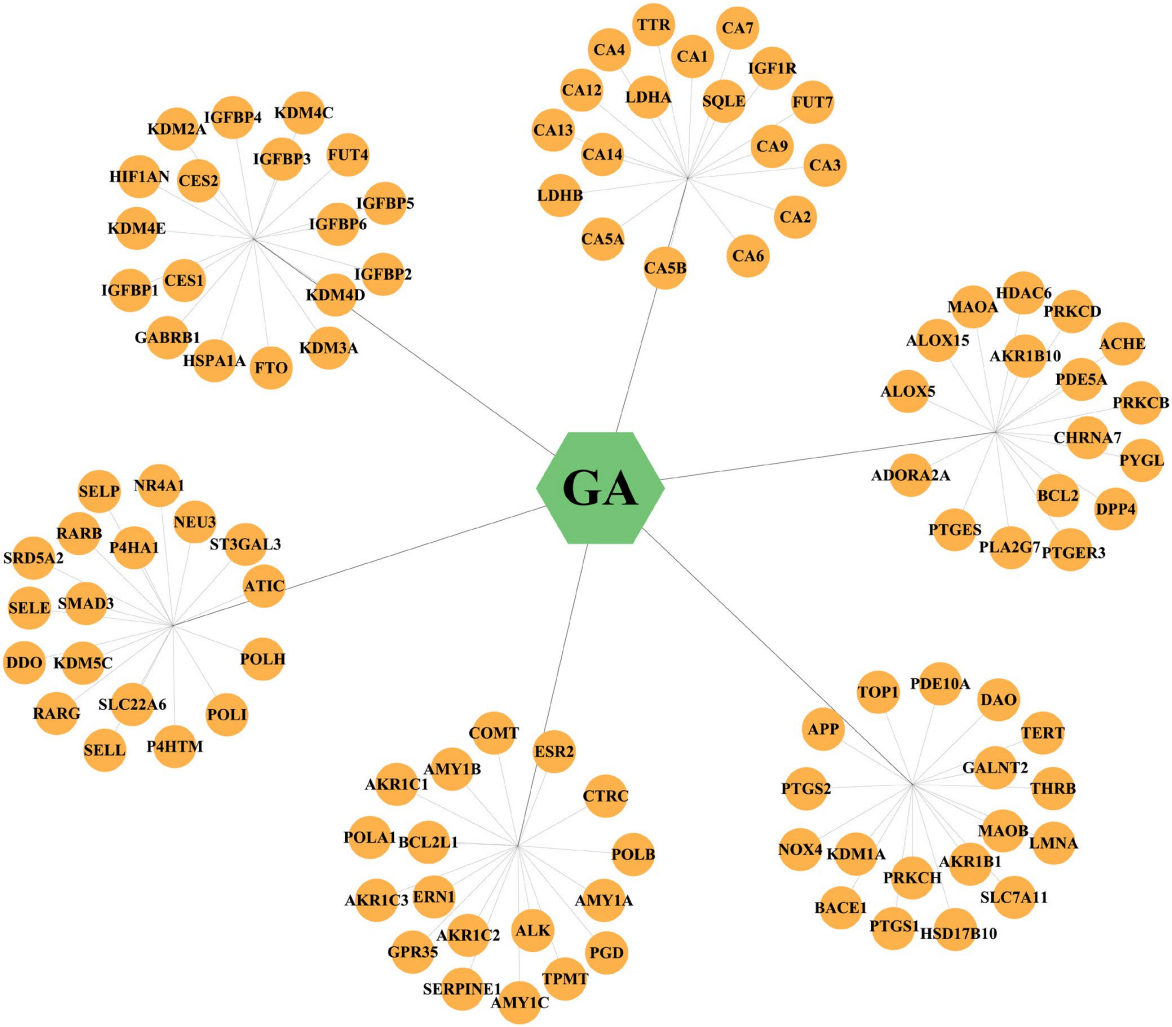
GA–target interaction network.

**FIGURE 4:**
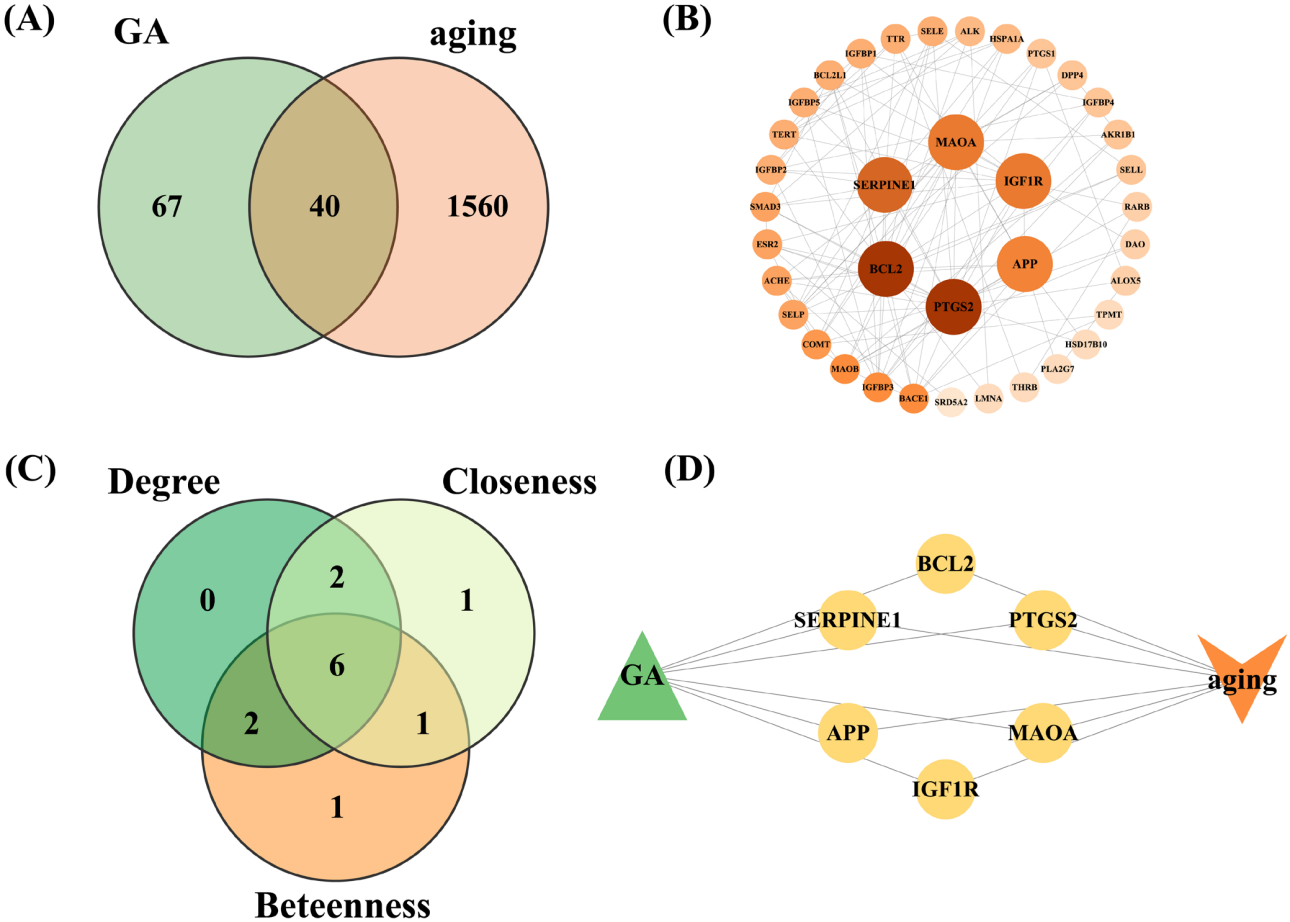
(A) Venn diagram of predicted GA targets and aging‐related targets. (B) PPI network of 40 overlapping targets. (C) Venn diagram of the top 10 targets ranked by degree centrality (DC), betweenness centrality (BC), and closeness centrality (CC). (D) GA–target–disease network.

A total of 25, 66, 5, and 41 targets of GA were predicted using SwissTargetPrediction, SEA Search Server, Super‐PRED, and CODD‐Pred, respectively. After removal of duplicates, 107 unique targets were retained and used to construct the GA–target interaction network in Cytoscape 3.9.1 (Figure 3, Table S1). In addition, aging‐related targets were retrieved from multiple databases, including 1595 targets from GeneCards, 6 targets from OMIM, and 32 targets from MalaCards. After deduplication, 1600 aging‐related targets were retained (Table S2). Venn diagram analysis revealed 40 overlapping genes between predicted GA targets and aging‐related targets, which were identified as potential anti‐aging targets of GA (Figure 4A).

### 3.3 PPI Network Construction and Key Target Identification

The 40 overlapping targets were imported into the STRING database to generate the PPI network, which was visualized using Cytoscape (Figure 4B). The resulting network consisted of 37 nodes and 236 edges, with node color intensity reflecting degree centrality (DC) (Table S3). The CytoNCA plugin was used to compute DC, betweenness centrality (BC), and closeness centrality (CC). The top 10 genes from each metric were intersected to identify six core targets: MAOA, PTGS2, BCL2, APP, IGF1R, and SERPINE1 (Figure 4C; Table S4). These six genes were considered key anti‐aging targets and were used to construct the GA–core targets–disease network (Figure 4D).

### 3.4 GO and KEGG Pathway Enrichment Analysis

**FIGURE 5:**
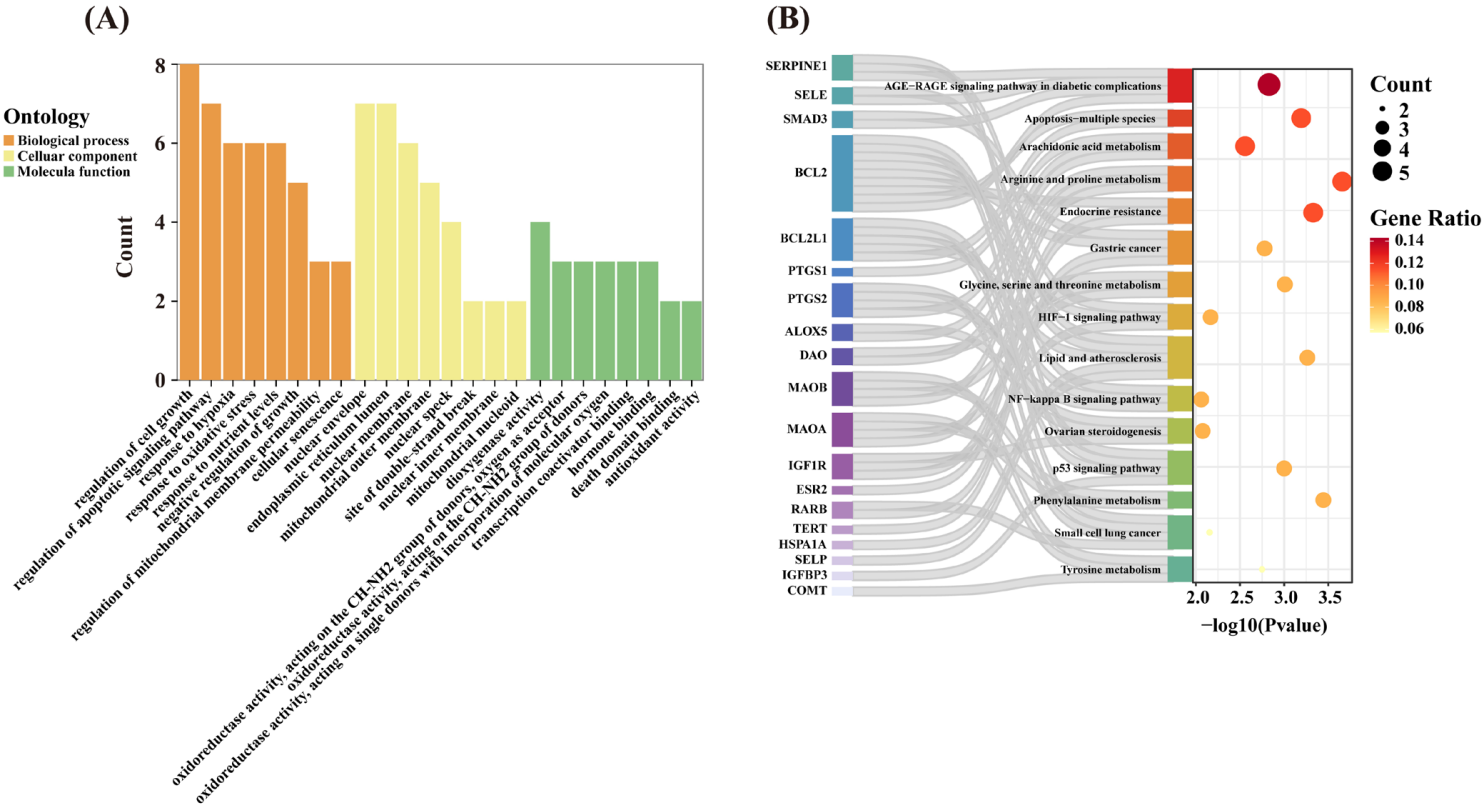
(A) GO enrichment analysis of overlapping targets. (B) KEGG enrichment analysis of overlapping targets.

GO enrichment analysis (Figure 5A) revealed that the six core targets were significantly enriched to 439 BP, 26 CC, and 51 MF, accounting for 84.91%, 5.03%, and 9.86% of the total, respectively (Table S5, *p* < 0.05). Key BPs included regulation of cell growth, regulation of apoptotic signaling pathway, negative regulation of growth, and cellular senescence. Enriched CCs were primarily associated with nuclear structures (nuclear envelope, nuclear inner membrane, nuclear speck), mitochondrial components (mitochondrial outer membrane, mitochondrial nucleoid), and cellular stress repair systems (nuclear membrane, site of double‐strand break). Enriched MFs involved redox and antioxidant activities (oxidoreductase activity, acting on the CH‐NH2 group of donors, antioxidant activity) as well as transcriptional regulation and signal transduction (transcription coactivator binding, death domain binding).

KEGG pathway analysis revealed significant enrichment in 22 signaling pathways (Table S6, *p* < 0.05), including those closely associated with aging, such as the NF‐kappa B signaling pathway, HIF‐1 signaling pathway, p53 signaling pathway, and the AGE‐RAGE signaling pathway in diabetic complications (Figure 5B). These results suggest that the core targets regulate aging through oxidative stress modulation, metabolic dysfunction, and apoptotic pathways.

### 3.5 Molecular Docking

Molecular docking was performed to evaluate the binding affinity between GA and the six core targets. Docking scores for all six complexes were below −5.0 kcal/mol (Table 1), indicating strong binding affinity. MAOA–GA and PTGS2–GA showed the highest affinities (−6.8 kcal/mol), followed by BCL2–GA (−5.6 kcal/mol), APP–GA and IGF1R–GA (both −5.5 kcal/mol), and SERPINE1–GA (−5.0 kcal/mol). Compared with the reference drug metformin, *GA* showed stronger binding affinities for all targets.

**TABLE 1:** Molecular docking results of GA with core targets.

Target	Affinity (kcal/mol)	
	GA	Metformin
MAOA	−6.8	−5.3
PTGS2	−6.8	−5.5
BCL2	−5.6	−4
APP	−5.5	−4.5
IGF1R	−5.5	−4.4
SERPINE1	−5	−4.4

**FIGURE 6:**
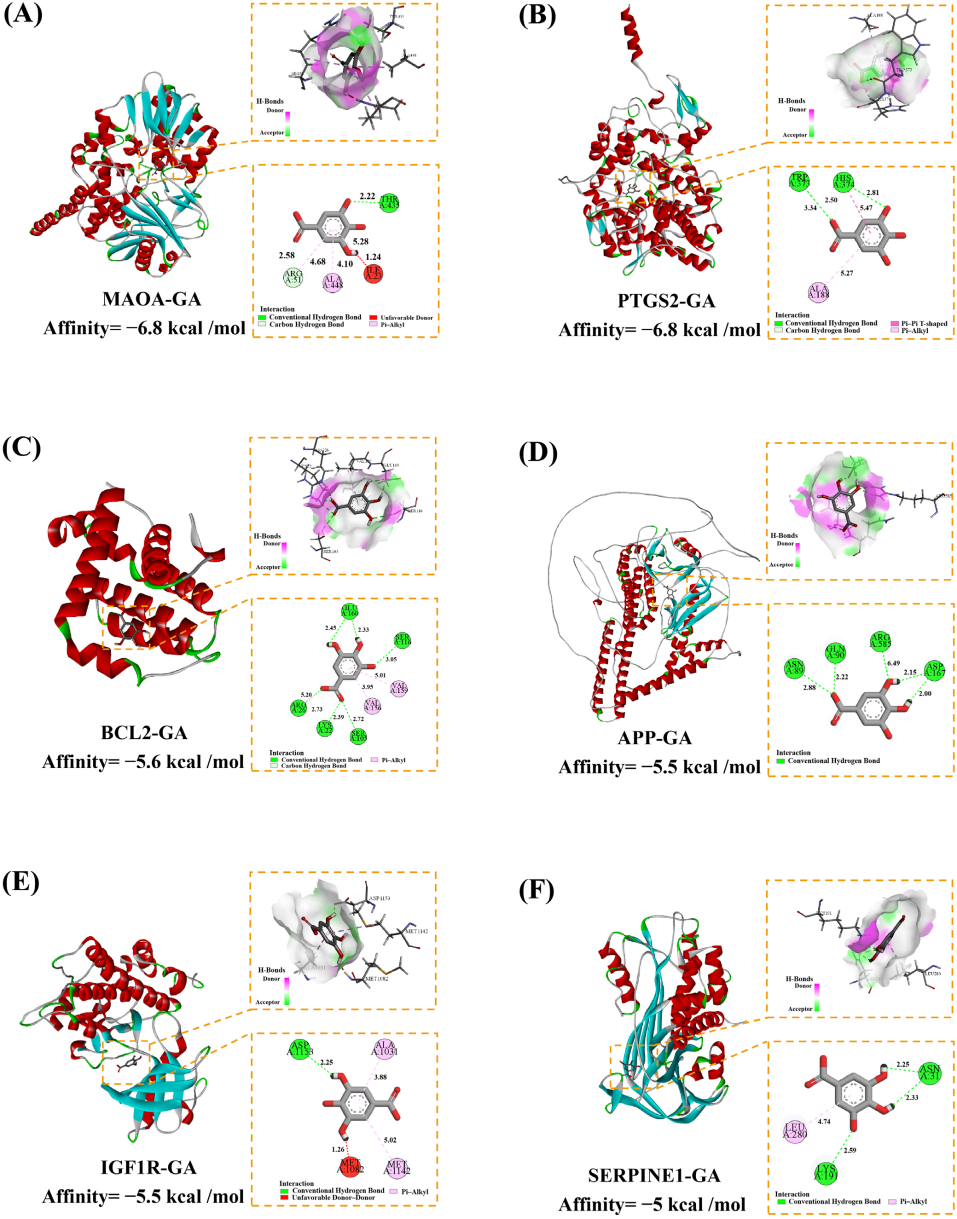
Molecular docking of GA with MAOA, PTGS2, BCL2, APP, IGF1R and SERPINE1. (A) Interaction diagram of MAOA with GA. (B) Interaction diagram of PTGS2 with GA. (C) Interaction diagram of BCL2 with GA. (D) Interaction diagram of APP with GA. (E) Interaction diagram of IGF1R with GA. (F) Interaction diagram of SERPINE1 with GA.

The specific binding interactions between GA and the six key anti‐aging targets—MAOA, PTGS2, BCL2, APP, IGF1R, and SERPINE1—are illustrated in Figure 6. In all cases, GA was tightly accommodated within the binding pockets of the target proteins and stabilized through a combination of hydrogen bonding and hydrophobic interactions (Table S7). In MAOA, hydrogen bonds were formed with THR435 (2.22 Å) and ARG51 (2.58 Å), while hydrophobic interactions involved ILE23 (5.28 Å), ARG51 (4.68 Å), and ALA448 (4.10 Å). In PTGS2, GA established hydrogen bonds with TPR373 (3.34 Å) and HIS374 (2.81 and 2.50 Å), and hydrophobic contacts with HIS374 (5.47 Å) and ALA188 (5.27 Å). In BCL2, multiple hydrogen bonds were observed with GLU160 (2.33 and 2.45 Å), LYS22 (2.39 and 2.73 Å), ARG26 (5.20 Å), SER105 (2.72 Å), and SER116 (3.05 Å), along with hydrophobic interactions involving VAL156 (3.95 Å) and VAL159 (5.01 Å). In APP, GA formed hydrogen bonds with ASP167 (2.00 and 2.15 Å), ASN89 (2.88 Å), and GLN90 (2.22 Å), and a hydrophobic interaction with ARG585 (6.49 Å). In IGF1R, one hydrogen bond was established with ASP1153 (2.25 Å), accompanied by hydrophobic interactions with ALA1031 (3.88 Å) and MET1142 (5.02 Å). In SERPINE1, GA formed hydrogen bonds with ASN31 (2.33 and 2.25 Å) and LYS191 (2.59 Å), as well as a hydrophobic interaction with LEU280 (4.74 Å). These molecular interactions indicate that GA binds effectively and specifically to each of the six targets, contributing to its predicted anti‐aging activity.

### 3.6 Molecular Dynamics Simulation and Binding Free Energy Calculations

**FIGURE 7:**
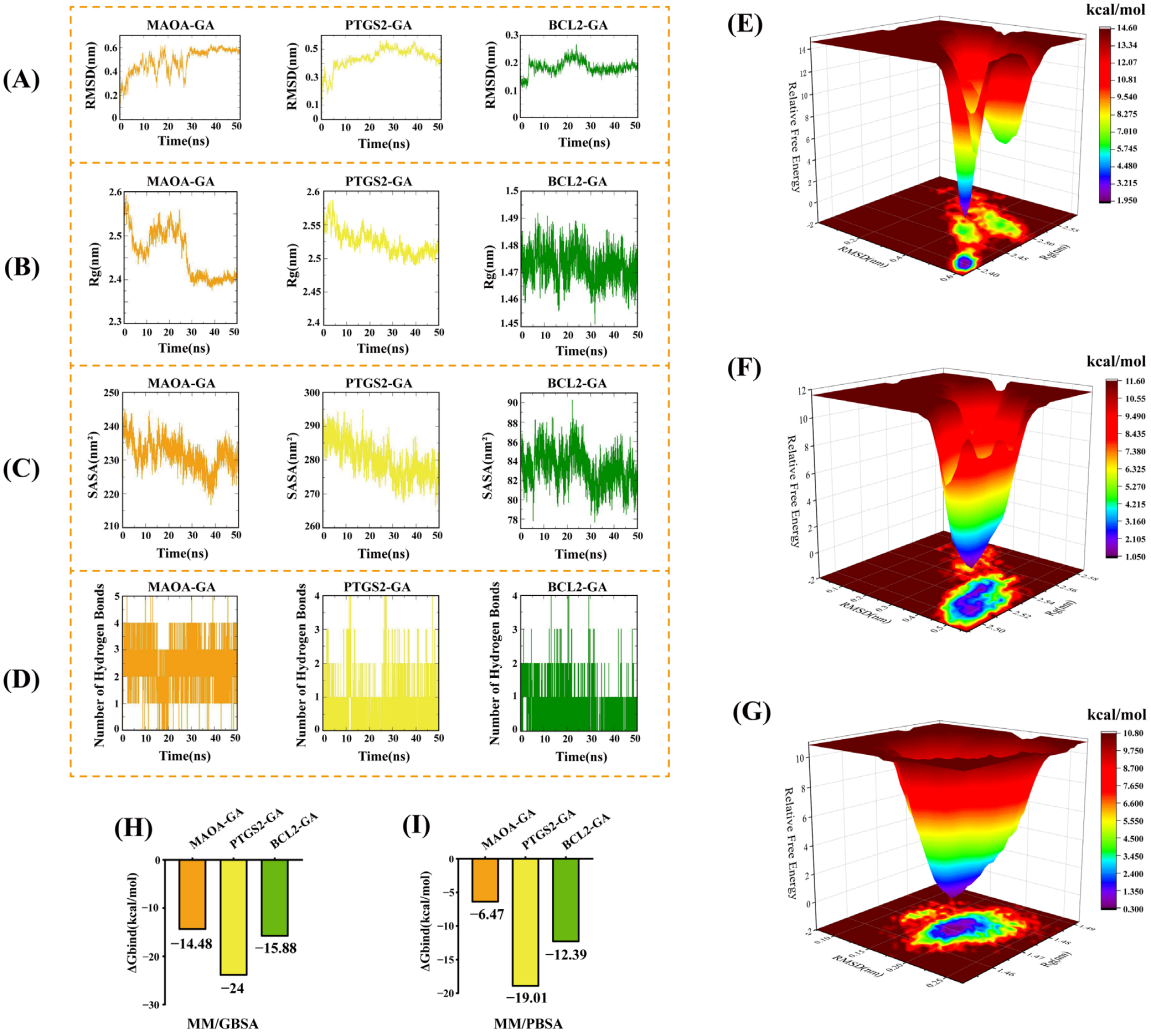
(A) RMSD values of the three complexes. (B) Rg values of the three complexes. (C) SASA values of the three complexes. (D) Number of hydrogen bonds in the three complexes. (E) ΔG_bind_ of the three complexes calculated using the MM/GBSA method. (F) ΔG_bind_ of the three complexes calculated using the MM/PBSA method. (G) Free energy landscape of the MAOA–GA complex. (H) Free energy landscape of the PTGS2–GA complex. (I) Free energy landscape of the BCL2–GA complex.

To assess the stability of protein–ligand interactions, MD simulations were conducted for MAOA–GA, PTGS2–GA, and BCL2–GA complexes. As shown in Figure 7A, RMSD values quickly stabilized at 0.48 ± 0.10 Å, 0.44 ± 0.07 Å, and 0.18 ± 0.02 Å for the three complexes, respectively. Rg values also remained stable throughout the 50 ns simulation (Figure 7B), averaging 2.46 ± 0.05 nm, 2.52 ± 0.02 nm, and 1.47 ± 0.01 nm. Solvent‐accessible surface area (SASA), a key indicator of protein folding and stability, remained stable across the simulations at 231.58 ± 4.37 nm^2^ (MAOA–GA), 280.13 ± 5.16 nm^2^ (PTGS2–GA), and 83.28 ± 1.82 nm^2^ (BCL2–GA) (Figure 7C). Hydrogen bond analysis showed that MAOA–GA formed the most stable and abundant interactions, followed by BCL2–GA and PTGS2–GA (Figure 7D). Free energy landscapes were constructed using RMSD, Rg, and Gibbs free energy as X, Y, and Z axes, respectively (Figure 7E–G). The lowest‐energy conformations (purple regions) represented the most stable structures. Binding free energy was further evaluated using MM/GBSA and MM/PBSA methods. As shown in Figure 7H, MM/GBSA‐derived ΔG_bind_ values were −24 kcal/mol (PTGS2–GA), −15.88 kcal/mol (BCL2–GA), and −14.48 kcal/mol (MAOA–GA). MM/PBSA values showed a similar trend (Figure 7I): −19.01 kcal/mol, −12.39 kcal/mol, and −6.47 kcal/mol, respectively. Lower ΔG_bind_ values indicate stronger binding affinity [31], supporting the conclusion that GA binds tightly to MAOA, PTGS2, and BCL2.

## 4 Discussion

Aging, characterized by the progressive decline of physiological functions with age, is a major risk factor for numerous chronic diseases and a growing global public health concern (Wang et al. 2024). In recent years, the pharmacological effects of white tea and its primary phenolic component, GA, have been increasingly investigated, supporting its potential as a natural anti‐aging agent. Multiple studies have reported that GA exerts significant antioxidant and anti‐inflammatory effects, which may contribute to its anti‐aging activity (Yan et al. 2018). In this study, the temporal variation in GA content across white tea samples aged for different years was systematically assessed, and the underlying anti‐aging mechanisms of GA were explored using network pharmacology and computational simulations.

The findings revealed a significant increase in GA content with prolonged storage time, in agreement with previous studies (Wu et al. 2023). DPPH, ABTS, and FRAP assays were used to evaluate antioxidant capacity by assessing the ability to scavenge lipid‐soluble radicals, scavenge water‐soluble radicals, and reduce ferric ions, respectively (Wang, Wang, et al. 2023). The APC integrated these three indices using a membership function approach, offering a comprehensive measure of antioxidant capacity (Liang et al. 2021). Both white tea samples and GA standard solutions showed significantly enhanced APC values with increasing storage years and GA concentrations. These results indicate that the aging process of white tea promotes GA accumulation, thereby enhancing overall antioxidant capacity, which may contribute to its anti‐aging potential.

Furthermore, a “GA–target–disease” network was constructed via network pharmacology to identify key targets and mechanisms involved in GA's anti‐aging effects. PPI network analysis identified six potential core targets: MAOA, PTGS2, BCL2, APP, IGF1R, and SERPINE1, all of which may be implicated in aging regulation. MAOA‐dependent oxidative stress has been shown to induce mitochondrial damage, telomere‐associated foci formation, and senescence markers in MAOA‐overexpressing transgenic mouse models (Martini et al. 2021). PTGS2 expression is elevated during intervertebral disc cell senescence, and its inhibition has been reported to alleviate aging and slow intervertebral disc degeneration (Li and An 2023). BCL2 is a well‐known anti‐apoptotic protein that prevents programmed cell death (Liu et al. 2018). APP contributes to age‐related neurodegenerative changes by disrupting the expression of synapse‐related genes via histone deacetylase (HDAC) regulation, thereby accelerating memory loss in Alzheimer's disease (McClarty et al. 2023). IGF1R plays a dual role in cardiac health, promoting physiological hypertrophy in early stages but impairing cardiac function during later stages by inhibiting autophagy and oxidative phosphorylation (Abdellatif et al. 2022). SERPINE1 overexpression has been linked to enhanced cellular senescence and increased levels of *β*‐galactosidase activity, oxidative stress, and DNA damage markers in intrauterine inflammation‐induced lung injury models (Yao et al. 2022). These findings suggest that GA may exert anti‐aging effects by modulating these six targets involved in diverse cellular aging pathways.

GO and KEGG enrichment analyses further illuminated the mechanisms by which GA may regulate aging. GO analysis indicated that GA was involved in key biological processes such as response to oxidative stress, metabolic regulation, apoptotic signaling, redox reactions, and antioxidant functions. KEGG pathway analysis revealed enrichment in several aging‐related pathways, including NF‐*κ*B, HIF‐1, and p53 signaling, as well as the AGE–RAGE signaling pathway involved in diabetic complications. Oxidative stress is a central driver of cellular damage, accelerating aging and increasing susceptibility to age‐related diseases (Vatner et al. 2020). NF‐*κ*B is a key transcriptional regulator of genes associated with inflammation and oxidative stress (Roy et al. 1996). HIF‐1 is a critical transcription factor in the cellular hypoxia response, capable of activating antioxidant gene expression and mitigating oxidative damage (Datta Chaudhuri et al. 2020). p53 modulates oxidative stress through regulation of downstream antioxidant genes such as ALDH4 and GPX1, thereby protecting cells from reactive oxygen species (ROS)‐induced damage (Y. Chen et al. 2018; Yoon et al. 2004). AGEs can alter the structure and function of proteins, lipids, and nucleic acids, and by interacting with the receptor RAGE, trigger oxidative stress and inflammation—processes that promote aging‐related diseases (Yamagishi and Matsui 2018). Notably, crosstalk between RAGE and p53 has been observed in senescent preadipocytes (Chen et al. 2014). Collectively, these results suggest that GA may counteract aging by modulating oxidative stress, inflammation, and metabolic dysregulation through multiple signaling pathways.

Computational simulations were employed to further investigate the binding affinity and mechanisms between GA and the identified aging‐related targets. Molecular docking revealed that all six protein–ligand complexes had binding energies below −5.0 kcal/mol, indicative of strong binding interactions (C. Li et al. 2022). Furthermore, GA showed stronger binding affinities with these targets than the reference drug metformin.

Molecular dynamics simulations were then conducted to assess the time‐dependent stability of the top three complexes (MAOA–GA, PTGS2–GA, BCL2–GA). RMSD values indicated that each complex achieved a stable conformation during the simulation period. Rg analysis confirmed tight receptor–ligand binding, while SASA values reflected protein folding stability. The number of hydrogen bonds formed was consistent with binding strength, with MAOA–GA showing the highest hydrogen bond density (Barazorda‐Ccahuana et al. 2020; Sarker et al. 2023). These structural stability parameters indicated that all three complexes were compact and conformationally stable. Additionally, binding free energy calculated using MM/GBSA and MM/PBSA methods confirmed the strong binding capabilities of GA to MAOA, PTGS2, and BCL2, thereby validating the molecular docking results.

## 5 Conclusion

In this study, the evolution of GA in aged white tea was systematically analyzed, and its potential anti‐aging mechanisms were explored using network pharmacology and computational simulation. The results showed that GA content increased progressively with storage years across seven white tea samples, and the APC increased in parallel with GA content. Network pharmacology analysis identified MAOA, PTGS2, BCL2, APP, IGF1R, and SERPINE1 as key anti‐aging targets of GA. Among the enriched pathways, those related to oxidative stress played a particularly important role in the anti‐aging effects of GA. Molecular docking and molecular dynamics simulations demonstrated that GA could effectively bind to MAOA, PTGS2, and BCL2 through hydrogen bonding and hydrophobic interactions, with stable binding conformations. These findings provide valuable insights into the functional transformation of GA during white tea aging and offer a theoretical basis for developing aged white tea or GA as a potential anti‐aging functional additive.

## Author Contributions

**Yanming Tuo:** conceptualization, writing – original draft, visualization. **Xiaofeng Lu:** formal analysis, investigation. **Qingying Song:** software, visualization. **Ruiqi Huang:** data curation, visualization. **Jiapeng Huang** and **Huan Sun:** data curation, formal analysis. **Yutao Shi** and **Ningkai Liao:** visualization, investigation. **Liangyu Wu** and **Jinke Lin:** writing – review and editing, project administration. **Yunfei Hu:** conceptualization, writing – review and editing, funding acquisition, project administration.

## Funding

This research was funded by the Science and Technology Backyard Project of Nanping City: Study on the Flavor and Quality of Zhenghe White Tea (KH240141A) and the Technology Innovation of Fujian Zhang Tianfu Tea Development Foundation (FJZTF01). The funders had no role in the design, determination, and interpretation of data, or in writing the manuscript.

## Conflicts of Interest

The authors declare no conflicts of interest.

## Supporting information


**TABLE S1:** Information for 107 potential targets of gallic acid.
**TABLE S2:** Aging‐related 1600 potential targets in 3 databases.
**TABLE S3:** PPI network of key targets.
**TABLE S4:** The top 10 targets of DC, BC, and CC.
**TABLE S5:** 22 signaling pathways from KEGG enrichment analysis.
**TABLE S6:** BP, CC, and MF from GO enrichment analysis.
**TABLE S7:** Molecular docking data of four key catechin components and core targets.

## Data Availability

The data that support the findings of this study are available from the corresponding author upon reasonable request.
